# RHUPUS Syndrome in Children: A Case Series and Literature Review

**DOI:** 10.1155/2013/819629

**Published:** 2013-05-15

**Authors:** Vahid Ziaee, Mohammad Hassan Moradinejad, Reyhaneh Bayat

**Affiliations:** ^1^Department of Pediatrics, Tehran University of Medical Sciences, Tehran, Iran; ^2^Pediatric Rheumatology Research Group, Rheumatology Research Center, Tehran University of Medical Sciences, Tehran, Iran; ^3^Division of Pediatric Rheumatology, Children's Medical Center, Pediatrics Center of Excellence, Tehran 14194, Iran; ^4^Growth and Development Research Center, Tehran University of Medical Sciences, Tehran, Iran

## Abstract

*Objective*. Overlap of juvenile idiopathic arthritis (JIA) and juvenile systemic lupus erythematosus (JSLE) is a rare clinical condition in children. This condition has been described as RHUPUS syndrome. Prevalence of this syndrome and 3 cases are reported in this paper. *Cases Presentation*. During 10 years, 3 patients with SLE had chronic arthritis before or after diagnosis of SLE. Prevalence of this disorder in JSLE was 2.5%. Two patients were females and one of them was a male. According to our review, mean delay between chronic joint involvement and JSLE diagnosis was 50.1 months. In our case report, two females had joint erosion and one of them died due to heart failure, but in the literature review, just 45% cases had joint erosion and 70% cases were polyarticulare form. *Conclusion*. RHUPUS is unusual presentation of lupus in children. It seems that clinical feature and outcome of RHUPUS syndrome are different in children due to difference between RA and JIA. We suggest juvenile RHUPUS for overlap of JIA and JSLE.

## 1. Introduction

Juvenile idiopathic arthritis (JIA) is the most common type autoimmune rheumatic disease in children. It is a chronic non infectious synovitis that can affect joints in any part of the body. Organs involvement is common in systemic onset type but it decreases after control of inflammation [1].

Systemic lupus erythematosus (SLE) is the most important, multisystem, autoimmune rheumatic disease characterized by both clinical and immunological abnormalities [2].

The peak incidence of childhood SLE occurs around puberty and predominantly involves young women in reproductive age, and in 10%–20% of SLE patients, diagnosis is made for the first time in childhood onset [2]. Arthritis is one of the earliest symptoms in juvenile SLE (JSLE) but it is characteristically short in duration and can be migratory [2]. Although the synovitis of JSLE may be minimally proliferative, it is only occasionally erosive and usually does not result in permanent deformity. JSLE can mimic JIA, especially when it is presented as chronic and erosive arthritis [3].

Overlapping JIA and JSLE is a rare clinical condition in children [4, 5]. RHUPUS syndrome has been defined as an overlap between RA and SLE in adult patients [6]. Although serological overlap between RA and SLE is common in adult patients, clinical overlap between RA and SLE is a rare manifestation of rheumatologic disorders. For the first time, RHUPUS syndrome was described by Schur in 1971 [7], but first clinical observation of this syndrome has been described in 1960 by Toone. He identified this clinical manifestation with this entity [8].

For diagnosis of RHUPUS syndrome, erosive polyarthritis and clinical symptoms of SLE as well as positive dsDNA or anti-Sm autoantibodies are necessary [9].

In this paper, we described the presence of three children with RHUPUS syndrome. Finally we reviewed all cases that have been reported in the literature with clinical picture of JIA as first presentation and eventually turned out to be typical systemic lupus erythemaosus, during follow-up.

## 2. Case Report

During 10 years, 120 patients with systemic lupus erythematosus who had been referred to our tertiary pediatric medical center were evaluated. Three patients (2 girls and 1 boy) had chronic joint involvement and JSLE (prevalence rate 2.5%). 

### 2.1. Case 1

 An 8 year-old girl was referred with pain, swelling, and motion limitation of both knees and left ankle lasting for more than 6 months. She has morning stiffness without fever. She did not have complaints in any other joints. On her first examination, she had bilateral effusions of the knees and swelling of left ankle, but the other joints and systems were normal. There were no skin rashes or mucosal lesions. Initial laboratory results revealed normal complete blood count (CBC), complement levels, and immunoglobulin levels. But RF and ANA were positive. In MRI, chronic inflammation with synovial thickening was seen in both knees, suggesting erosive arthritis. She was diagnosed as oligoarticular JRA and started treatment with prednisolone (7.5 mg/day), Methtrexate (7.5 mg/Week), and hydroxycholoroquine (200/mg/day), and she was regularly followed up at 1.5–2 months intervals. After two years of follow up, at the age of 10 years, she had a flare-up of disease with clinical manifestation (fever, fatigue, and malaise), malar rash, and arthritis in the knees, ankle, oral ulcer, pethecia, and purpura. At that time, laboratory results revealed (pancytopenia), coombs negative anemia (Hb: 9.7 g/dL), thrombocytopenia (30,000/mm3) and leukopenia (WBC count 2900/mm3) (predominant lymphopenia). F-ANA was positive (1/1280, speckled pattern), anti-dsDNA level was positive 1/80, rheumatoid factor level (RF) was 7.88 IU/mL (0–14 IU/mL), and C3 (65.7 mg/dL) and C4 (4.4 mg/dL) levels were low. Antiphospholipid antibodies (aPL) level was negative and urinalysis showed microscopic hematuria (5-10 RBC in hpf) and trace proteinuria (1+). She was diagnosed as JSLE, with more than five criteria, after two years of ongoing chronic arthritis. Treatment was started with new diagnosis of SLE with Prednisolone (1 mg/kg/day), Azathioprine (50 mg/d), and Hydroxycholoroquine (200/mg/day). She was under observation and she had a systemic flare-up of lupus when she was 12 years old which has been controlled with increasing dosage of drugs. When she was 13 years old, she found renal involvement in this course of the illness with clinical manifestations of SLE, hypertension, proteinuria, urine protein 24 hours more than 2300 mg/day, microscopic hematuria, cellular casts, and elevated serum blood nitrogen and creatinine concentration.

She was treated with azathioprine (50 mg/d) with oral prednisolone (50 mg/day) hydroxycholoroquine (400/mg/day). After 4-year followup, she is 17 years old and she is under observation and she was on low dose prednisolone (0.5 gr/kg/day), azathioprine (50 mg/d), and hydroxycholoroquine (400/mg/day).

### 2.2. Case 2

A 10-year-old girl was referred with morning stiffness and arthralgia in the both knees and ankles. In physical examination, she had sometimes fever, but there was not effusion and swelling in the joints or other systemic manifestation of rheumatologic disorders. In laboratory investigation, she had normal CBC, elevated ESR (40), and positive ANA (1/80). Other tests such as Anti ds-DNA, RF, VDRL, Urinalysis, BUN, and Creatinine were negative. She was under observ for 6 months and she was on naproxen 10 mg/kg during this time. After 6 months, joint effusion in both knees, ankles, and wrists was added to her symptoms. She was diagnosed as JRA polyarticuler form and she was treated with prednisolone (7.5 mg/day), Methtrexate, (7.5 mg/Week), and hydroxycholoroquine (100/mg/day) as well as Naproxane, and she was under observation every 2 months. When she was 12, she was referred to emergency clinic with chief complaint of abdominal pain and acute anemia and splenomegalia. Joint involvement in large joints (knees, elbow, and ankles) and small joints (MCPs and PIPs) was there. In laboratory study, we found an acute hemolytic anemia (Hgb = 5.2, Retic = 6%) with positive direct coombs (2+), positive VDRL (1.32), decreased complements (C3 = 21, C4 = 3, CH50 = 0%), and positive ds-DNA (100). She was diagnosed as an overlap syndrome (RAPUS syndrome) between JRA and JSLE, with more than four criteria, after 24 months ongoing chronic arthritis. Treatment was started with new diagnosis SLE with Prednisolone (2 mg/kg/day) and Hydroxycholoroquine (200/mg/day). After 1 month, anemia was resolved and joint symptoms reduced. During followup, she had multijoints involvement, when we reduced the dosage of prednisolone to less than 0.5 mg/kg/day. 

After 2 months, she was referred to clinic with abnormal movement without history of streptococcal infection. In physical examination, cardiac examination was normal and there was not any abnormality in neurologic examaination exept chorea athetosis. Laboratory investigation showed elevation in anti ds-DNA titer, decrement of complements, and normal MRI. The dosage of prednisolone was increased to 2 mg/kg/day and haloperidol and Artan was started. After 2 weeks, abnormal movement was controlled and the dosage of prednisolone was decreased after 4 weeks. She had a relapsing in chorea athetoeid movement after 3 months, and reevaluation did not show any new findings. After 12-month followup, she was under control with prednisolone (0.5 mg/kg/day), Hydoxychlroquine (200 mg/daily), valporate sodium, and haloperidol, but she had on and off joints effusion and in radiographic evaluation, and there is some evidence to erosion in her knees.

After 5 months, she was admitted to hospital with fever (39.5), vomiting and tachypnea, and large joints effusion. Treatment with antibiotics was started based on diagnosis of flare-up of disease because of infection. In second day, the decreased levels of consciousness, low cardiac output, GI bleeding, and anuria were presented. In heart echocardiography, she had ejection fraction: 10%, left ventricle, transverse diameter systole: 10%, dilated cardiomyopathy without pericardial effusion and/or vegetation. Blood culture was negative, and after starting pulse therapy with methylprednisolone, she died due to heart failure.

### 2.3. Case 3

An 11-year-old boy was referred with pain and swelling of right knee and swelling in the back of the ankle for 2 months, with no abnormal findings in physical exam. In laboratory investigation, he had anemia (HB: 8.8 and thrombocytosis (PLt: 569000), elevated ESR (59) and CRP (3+) and positive RF (3+). The patient's brother and sister were known lupus cases, and his brothers were died due to SLE complication; so investigations for finding lupus were done, and in laboratory tests, he had negative FANA and normal ds-DNA. He was treated with methotrexate, prednisolone (2.5 mg daily), and hydroxychloroquine (100 mg/day). After one year, he had a flare-up with no systemic problems and/or abnormal laboratory tests. In third year, he was in full remission and treatment was stopped. After 2 years of followup, he found joint effusion and mallar rash and positive FANA (1/320) and Anti ds-DNA. Regarding positive family history, diagnosis of JSLE was suggested and he was treated with prednisolone (5 mg TDS), hydroxychloroquine (100 mg daily) and azathioprine (1/2 tab daily). There is no evidence of joint erosion in radiologic evaluation. His symptoms were controlled after 2 months and the dosage of treatment was reduced, gradually. After 2 years of followup, he was 18 and he had no problems and was in remission.

## 3. Discussion

RHUPUS (RA and SLE) is a rare clinical condition in adult. First description of RHUPUS was done by Toone in 1960 [8]. In SLE, joint involvement may range from minor joint pain to severe deforming arthritis as well as Jaccoud's arthropathy, but sometimes joint pain is more severe than joint objective findings [13–15]. The diagnosis of RHUPUS is made when it becomes difficult to support the diagnosis of any of these diseases as an isolated entity. There are three viewpoints on RHUPUS. Some authors such as Fernandez believe that RHUPUS arthropathy is a serious articular involvement of lupus and it is different from a superposition or overlap of RA and SLE [16, 17], while other authors believe that RHUPUS syndrome is combination of SLE and chronic erosive polyarthritis [5, 9, 15], and the last group believes that RHUPUS is overlap of RA or JIA and SLE (regardless to poly or oligoarthritis and erosive or nonerosive) [4, 18]. We agree with last group about RHUPUS syndrome in children. Since, RHUPUS is overlap of RA or RA and SLE, we suggested juvenile RHUPUS for overlap of JIA and JSLE. RHUPUS in children presents with features of JRA but later on develops features of SLE [4]. Although, few cases of RHUPUS syndrome present simultaneously with features of JRA and SLE as overlap syndrome, JRA and SLE as a presenting manifestation may not be complete [5]. In adult the majority polyarticular erosive arthritis is most common manifestation, but in children, features of JIA dominating asymmetrical erosive and/or nonerosive oligoarticular involvement may be common manifestation [10, 11]. All cases with RHUPUS syndrome in this study and previous report in children were compared in Table 1. In this case report, two cases were oligoarticular subtype and one of them had joint erosion. Another case was polyarticular subtype with joint erosion. In the literature review, 70% of cases were polyarticular subtype.

Panush et al. in 1988 in an observational study reviewed approximately 7000 new patients who were evaluated for more than over 11 years [6]. They found 6 patients who had both RA and SLE (the prevalence 0.09%). However, it seems that the prevalence of this presentation of SLE is more common than previously reported. In our study, the prevalence of this syndrome was 2.5%. Similarly, Cavalcante et al. reported the prevalence of chronic polyarthritis in 2.6% of JSLE patients and in all patients, joint involvement was the initial manifestation [5]. It can be due to better management and decreases in mortality of SLE during recent decades. 

There are few reports on RHUPUS syndrome in children [10–12]. Martini et al. reported an 11-year-old girl who presented initially as JRA patient at 5 years old, but she developed clinical manifestation of SLE after 6 years [12]. Similarly, Unsal et al. reported an 8.5 year old girl with JIA who developed criteria of SLE after 2 years [10]. In another report, Takei et al. introduced two cases with JRA who developed SLE criteria and after 8 years with ANA and anti ds-DNA positivity and clinical picture of SLE [11]. Our cases fulfilled JSLE criteria after 2-3 years of first manifestation of joint involvement and JIA diagnosis. The mean delay of SLE diagnosis in all reported cases was 50.1 months (Table 1). 

Most common features of SLE in our cases were cutaneous involvement in 2 patients and hematologic manifestations in one case, but all of them had ANA and RF positivity before SLE diagnosis. 

Similar to our study, in most cases with RHUPUS syndrome, ANA and RF have been positive or they get positivity during followup [11, 19]. Similar with Panush report, our patients have increased frequency of positivity in high titers of ANA. Totally, the rate of positive ANA and RF at the onset of chronic arthritis was 50% and 60%, respectively (Table 1). 

It seems that articular erosion is less common in children than adult with RHUPUS syndrome. In our case report, one case had erosive arthritis before completing JSLE, and in our litrature review, just 45% cases had joint erosion (Table 1). Recently, Amezcua-Guerra studied the prevalence of antibodies against anti-CCP antibodies in RHUPUS [18], and they concluded that RHUPUS is an overlap between RA and SLE. 

## 4. Conclusion

Overlap of JIA and JSLE is a rare syndrome in childhood and there is a few case report on this syndrome in children. It seems that clinical feature and outcome of RHUPUS syndrome are different in children due to difference between RA and JIA. Juvenile RHUPUS is suggested for overlap of JIA and JSLE. We recommend all patients with JRA and positive ANA to have followup for long time (at least 4 years) for Juvenile RHUPUS syndrome and to be evaluated for JSLE, if they developed cutaneous or hematological symptoms. 

## Figures and Tables

**Table 1:** Clinical and paraclincal features and outcome of cases with RHUPUS syndrome in this case report and previous reports in children.

Report	Sex	JIA Characteristics					Delay in SLE diagnosis (mo)^†^
		Age at onset (y)	ANA	RF	Subtype	Erosion	
Unsal et al. [10]	Female	8.5	−	−	Oligoarticular	No	24
Bazsó et al. [4]	Female	12	−	−	Polyarticular	No	120
	Female	10	+	+	Polyarticular	No	11
Cavalcante et al. [5]	Female	10	+	+	Polyarticular	Yes	9
	Male	5	+	+	Polyarticular	Yes	15
Takei et al. [11]	Female	15	−	−	Polyarticular	No	96
	Female	3	−	−	Polyarticular	?	120
Martini et al.* [12]	Female	5	?	?	?	?	72
	Female	8	+	+	Oligoarticular	Yes	24
Our study	Female	10	+	+	Polyarticular	Yes	24
	Male	11	−	+	Oligoarticular	No	36

Total cases	82% female	8.1	50% positive	60% positive	70% polyarticular	45% erosion	50.1

*We did not access full text of this report.

^†^Delay between beginning of chronic arthritis and SLE diagnosis.
